# Prevalence of latent tuberculosis infection in Sudan: a case–control study comparing interferon-γ release assay and tuberculin skin test

**DOI:** 10.1186/1471-2458-13-1128

**Published:** 2013-12-05

**Authors:** Amani Osman Shakak, Eltahir Awad Gasim Khalil, Ahmed Mudawi Musa, Kawthar Abd Eljalil Mohamed Salih, Abd Elgadir Ali Bashir, Ala Hassan Ahmed, Fath Elrahman Mohamed Idris, Ahmed Mohamed Elhassan

**Affiliations:** 1 Faculty of Medical Sciences, University of Shendi, Shendi, Sudan; 2 Department of Clinical Pathology & Immunology, Institute of Endemic Diseases, University of Khartoum, Medical Campus, Qasr Avenue, P.O. Box 45235, Postal code 11111, Khartoum, Sudan; 3 Elneil Medical College, Khartoum, Sudan; 4 Medical Commission, Khartoum State Ministry of Health, Khartoum, Sudan; 5 Department of Medicine, Faculty of Medicine, University of Khartoum, Khartoum, Sudan; 6 Khartoum North Teaching Hospital, Khartoum North, Sudan; 7 Central Laboratory, Ministry of Science & Communications, Khartoum, Sudan

**Keywords:** Latent Tb infection, IFN-γ release assay, TST, Sudan, Contact tracing

## Abstract

**Background:**

Most people exposed to *M. tuberculosis* show no evidence of clinical disease. Five to 10% of individuals with latent infection progress to develop overt disease during their life time. Identification of people with latent TB infection will increase case detection rates and may dictate new treatment policies to control tuberculosis. This study aimed to determine LTBI point prevalence in a population from Sudan using two different diagnostic methods: the tuberculin skin test (TST) and the IFN-γ release assay (IGRA).

**Methods:**

This was a prospective, community-based and case-controlled study. Following informed consent, household contacts (HHCs; n = 98) of smear-positive index cases and Community controls (CCs; 186), were enrolled. Tuberculin skin test (TST), whole blood stimulation with ESAT-6/CFP-10 ± TB7.7 antigens or purified protein derivative (PPD) and IFN-γ levels determination with ELISA were performed. The levels of IFN-γ and TST induration between the CCs and the HHCs were compared using student *t*-test, Chi-square and Kappa coefficient. Pearson correlation test was used to compare TST and IFN-γ. *P* levels of <0.05 were considered significant.

**Results:**

TST induration of ≥ 10 mm gave an LTBI point prevalence of 327 cases/1000 individuals among HHCs compared to 126 cases/1000 individuals among CCs (*p =* 0.000). PPD-induced IFN-γ release assay gave an LTBI point prevalence of 418 cases/1000 individuals among HHCs compared to 301 cases/1000 individuals among CCs *(p =0.06).* On the other hand ESAT-6/CFP-10 ± TB7.7-induced IFN-γ gave an LTBI point prevalence of 429 cases/1000 individuals among HHCs compared to 268 cases/1000 individuals among CCs (*p* = 0.01). IFN-γ productions levels induced by ESAT-6/CPF-10 ± TB7.7 antigens in HHCS and CCs were not significantly different from those induced by PPD (*p* = 0.7).

**Conclusion:**

IFN-γ release assay (IGRA) gave higher LTBI point prevalence compared to TST in HHCs and CCs. PPD gave comparable results to ESAT-6/CFP-10 ± TB7.7 antigens in whole blood IFN-γ release, making it a cheap alternative to the recombinant antigens.

## Background

Tuberculosis (TB) is a contagious and airborne disease of poverty that mostly affects young adults in their most productive years in developing countries. The vast majority of TB deaths are in the developing world with an estimated two million deaths annually. WHO reported that one third of the world population is infected with latent TB with 9.4 million new overt cases annually [1]. Five to 10% of individuals with latent TB progress to develop overt disease during their life time. Identification of people with latent TB infection (LTBI) will increase case detection rates and may dictate new treatment policies to control tuberculosis. In the absence of a gold standard test for identification of individuals with LTBI, different reports suggested tuberculin skin test (TST) or interferon-γ release assay (IGRA) as the best test [2-7].

The tuberculin skin test (TST) remains the most commonly used test for diagnosing latent TB infection (LTBI) that could indicate preventive treatment [2]. TST has a number of limitations: low sensitivity in immune-compromised patients and cross-reactivity with hypersensitivity in BCG-vaccinated and non-tuberculous mycobacterial (NTM) infections [3]. Recently a new diagnostic test IFN-γ release assay (IGRA) which measures the production of IFN-γ in whole blood upon stimulation with specific antigens has been introduced. These antigens (Early Secreted Antigenic Target-6; ESAT-6 and Culture Filtrate Protein-10; CFP-10), though unique to *M. tuberculosis* in the latent state, have been found in some non-tuberculous *Mycobacteria* and *M. leprae*. IFN-γ produced by T-cells plays a critical role in protective immunological responses to primary infection. The QuantiFERON-TB Gold In-Tube (QFT-GIT; Cellestis Ltd. Carnegie, Victoria, Australia) is the newest IGRA assay. In addition to the ESAT-6 and CFP-10 peptides, this test utilizes the TB7.7-*M. tuberculosis* specific peptide as well. Unfortunately, IGRAs cannot differentiate between active and past TB infections or between LTBI and tuberculosis [4-6].

This study aimed to determine LTBI point prevalence in a Sudanese population using TST and the IFN-γ release assay. The study also aimed to compare PPD and ESAT-6/CPF-10 ± TB7.7 antigens as stimulants for IFN-γ release in whole blood stimulation in vivo systems.

## Methods

### Scientific and ethical considerations

The study was approved and passed by the Ethics & Scientific Committees of the Institute of Endemic Diseases, University of Khartoum and the Ethics Committees of the Federal Ministry of Health, Khartoum. Written informed consents were obtained from all participating individuals. The national TB control program was heavily involved from the start of the study.

### Study population

Index cases with smear-positive sputum pulmonary TB and at least one household contact were initially enrolled. House hold contacts (HHCs) of these cases were recruited based on the following inclusion criteria: age ≥15 years, living for more than six months at the same property as the respective index case, agreeing to participate and they/their guardian sign the informed consent. Sex-matched Community controls (CCs) were enrolled if: age ≥15 years, have no history of tuberculosis in his/her household, have no symptoms/signs of tuberculosis and has signed an informed consent.

### Tuberculin skin testing

Tuberculin skin test (TST) was performed on all contacts by the administering 0.1 ml containing 5 tuberculin units (Razi Institute, Iran). Transverse induration was measured 72 hours later using the ballpoint pen technique. A reaction equal or more than 5 mm was considered as reactive [8].

### Whole blood stimulation and IFN-γ release assay

For one hundred individuals whole blood stimulation and measurement of IFN-γ levels was conducted using QFT-G- in Tube Kit (Cellestis Ltd., Victoria, Australia) as per manufacturer’ instructions. Due to interruption of Kits supplies, *M. tuberculosis* recombinant antigens (ESAT-6/CFP-10, Cellestis Ltd., Victoria, Australia) and PPD were used in the rest of the volunteers (184 CCs), while IFN-γ production assay was performed using ELISA Kits from Komabiotech, Seoul, South Korea as per manufacturer’ instructions. The IFN-γ measurement kits were validated and compared at the QC Unit of the Institute of Endemic Diseases, University of Khartoum. Whole blood stimulation was performed as follows: one ml of blood was taken in each of four tubes: 1. ESAT-6/CFP-10 antigens 2. Phytohemagglutinin (PHA) for the positive control 3. No antigen for the negative control 4. Five PPD antigen units. The tubes were incubated for 24 hours at 37°C in Cellestis incubator (Cellestis Ltd., Victoria, Australia), and plasma was collected after centrifugation and stored at −20°C until assayed. A cut-off of 0.35 IU/mL for IFN-γ was applied.

### Statistical analysis

Statistical analyses were performed using Epidemiological Information (Epi Info) software version 3.4.3.0. The levels of IFN-γ cytokine and TST induration between the CCs and the HHCs were compared using student *t*-test and Chi-square. Pearson correlation test was used to compare TST and IFN-γ. *P* levels of <0.05 were considered significant. Kappa coefficient of agreement between TST and IGRA; PPD-induced IFN-γ release and ESAT-6/CFP-10 ± TB7.7-induced IFN-γ release for the study population was calculated as described by Cohen previously [9].

## Results

A total of 284 consenting volunteers were enrolled in the study, ninety eight were House-Hold Contacts (HHCs) for 25 Index Cases and 186 were Community controls (CCs) with a male: female ratio of 1:4. The mean age of HHCs was 30 ± 11.0 years and that of CCs was 29 ± 10.0 years. Females were predominant in both HHCs and CCs, where they were four times more than males. All volunteers had history of BCG vaccination that was confirmed in the majority (>85%) by presence of a scar. TST induration and positivity were significantly different in HHCs compared to CCs (p = 0,000) (Table 1). Sixty per cent of HHCs had TST induration of <5 mm compared to 83% in CCs. Volunteers with TST induration of ≥10 mm were seen more frequent among HHCs (25.5%) compared to CCs (9%). Volunteers with TST of *≥* 15 mm were mainly seen in house hold contacts *(p = 000).* Nineteen of CCs could not be traced for TST reading in screening (Table 1).

**Table 1:** Tuberculin skin test (TST) & Cytokines production in whole blood cultures [ESAT-6/CFP-10 & PPD] in the study population at screening

**Variables**	**House-Hold contacts**	Community controls**	***p value***
	**(*n = 98)***^***¤***^	***(n = 186)***	
TST *(mean ± SD)*	5 ±6	2 *±*3	0.000^*^
*< 5 mm*	59 (60.2%)	139 (83.2%)	0.000^*^
*5 - 9 mm*	12 (12.2%)	13 (7.8%)	0.04^*^
*10 - 14 mm*	13 (13.2%)*§*	14 (8.4%)	0.1
*≥ 15 mm*	14 (14.4%)*§*	01 (0.6%)	0.000^*^
**IFN-γ Mean level IU/ml:**			
*Purified Protein Derivative:*			
*Mean ± SD*	0.5 ± 0.6	0.3 ± 0.4	0.4
*< 0.35*	57/98 (58.2%)	130/186 (69.9%)	0.06
*0.35-1.0*	28/98 (28.6%)	44/186 (23.6%)	0.1
*1.1-2.0*	13/98 (13.2%)	12/186 (6.5%)	0.3
*ESAT-6 + CFP −10 ± TB7•7:*			
*Mean ± SD*	0.5 ± 0.7	0.2 ± 0.2	0.000^*^
<*0.35*	56/98 (57.1%)	136/186(73.2%)	0.03^*^
*0.35 - 1.0*	18/98 (18.4%)*§*	41/186 (22.2%)	0.01^*^
*1.1 - 2.0*	24/98 (24.5%)*§*	09/186 (4.6%)	0.2

Continuous variable are expressed as means ± SD. *Significant difference (p < 0.05).

§There was a low correlation between IFN-γ level >0.35 IU/ml and TST ≥10 mm (r = 0.4).

All volunteers had history of BCG vaccination that was confirmed in the majority (>85%) by presence of a scar. **Nineteen CCs did not report for TST reading.

The mean IFN-γ production level for the ESAT-6/CFP-10 ± TB7.7 whole blood stimulation was significantly different for the HHCs (0.5 ± 0.7 IU/ml) compared to the CCs (0.2 ± 0.2 IU/ml) (*p = 0.000).* These recombinant antigens stimulation gave IFN-γ production levels of ≥0.35 IU/ml in 42.9% (42/98) of HHCs compared to 26.9% (50/186) of CCs (*p* = 0.01) only in the 0,35-1,0 range. Fifty seven percent of the HHCs were not reactive in whole blood IFN-γ production compared to more than seventy percent in the CCs (*p = 0.03)*. In PPD-whole blood stimulation, IFN-γ production levels of ≥ 0.35 IU/ml were 41.8% (41/98) and 30.1% (56/186) for HHCs and CCs respectively (*p =* 0.06 not significant).

IFN-γ productions levels induced by ESAT-6/CPF-10 ± TB7•7 antigens were not significantly different from those produced by PPD in HHCs and CCs (*p* = 0.7). Kappa co-efficient of agreement between PPD-induced IFN-γ release and ESAT-6/CFP-10 ± TB7.7-induced IFN-γ release for the study population was calculated at 1 (Table 1).

No correlation between TST and IFN-γ production levels in HHCs and CCs could be detected (r = 0.4).

TST induration of ≥ 10 mm gave a LTBI a point prevalence of 327 cases/1000 individuals among HHCs compared to 126 cases/1000 individuals among CCs (*p =* 0.000). PPD-induced IFN-γ release assay gave an LTBI point prevalence of 418 cases/1000 individuals among HHCs compared to 301 cases/1000 individuals among CCs *(p =0.06).* On the other hand ESAT-6/CFP-10 ± TB7.7-induced IFN-γ gave an LTBI point prevalence of 429 cases/1000 individuals among HHCs compared to 268 cases/1000 individuals among CCs (*p* = 0.01). Overall, LTBI point prevalence was calculated in the study population for TST (induration ≥ 10 mm), PPD-IFN-γ induced and ESAT-6/CPF-10 ± TB7•7 assays at 453/1000, 719/1000 and 678/1000 respectively (Table 1).

The Cohen’s co-efficient of agreement between TST and IGRA for the study population was calculated at 0.52.

## Discussion

Tuberculosis control programs in developing countries are greatly haunted by low case detection rates and latent TB infections that constitute hidden pools that continuously feed new cases. Accurate diagnosis of LTBI and appropriate treatment of probable cases-to-be reduce the risk of progression to overt disease and increase case detection rates [7,10]. However the role of preventive treatment in high burden countries needs to be assessed. Furthermore, testing of adult contacts is controversial in high incidence countries [11]. At present time there is no gold standard test for the detection of individuals with LTBI and TST has been the most widely used diagnostic tool in the detection of LTBI for more than a century. TST is a simple and cheap test but has reduced specificity as BCG vaccination may affect the results as in our population. TST is an *in vivo* test that measures skin induration response due to cytokines/chemokines secretion (IL- 4, IFN-γ, tumor necrosis factor-α, IL-10, IL-12 and G-CSF) [2]. On the other hand, whole blood IFN-γ release assays are highly specific for latent *M. tuberculosis,* but are expensive, need special equipment and expertise and are not widely available in developing countries. In addition, these tests have limited predictive values to identify those patients who will develop overt tuberculosis [12-23]. IFN-γ release assay (IGRAs) measure only IFN-γ production, a T helper 1 response, so it remains possible that positive TST results might capture an immune response in truly infected individuals that are missed by the IFN-γ release assay [14].

In the absence of a gold standard test for LTBI, we took LTBI point prevalence as an objective measure of LTBI. In our study, IFN-γ release assays were more frequently positive in HHCs than the TST, a result that may indicate a better sensitivity. These data are in agreement with previous studies that reported that IFN-γ release assay is better than TST in detecting individuals with LTBI [12-22]. Lowering the TST induration to ≥ 5 mm gave an LBTI point prevalence that is comparable to IFN-γ release assay test in previous reports [20-22].

Low correlation between TST and IFN-γ release assay has been reported in this study as well as in many other studies [19,21,22]. On the other hand, many other studies have reported moderate to strong positive correlation between the TST and IFN-γ release assay tests. These later studies presented TST as the least cost-effective, while others reported that TST followed by IGRA as highly cost effective [22,24-26]. It is worth mentioning that a study from South Africa suggested that TST was more effective in identifying LTBI than three generations of a whole blood IFN-γ release assay tests in countries with high TB burden [27]. Park and colleagues found that the cost effectiveness of the different testing strategies for LTBI was very variable especially between tests reagents [28]. The cost of QFT (IGRA) per person was reported to be between $20 and $30, while that of TST was ~ $5. In our setting, the cost of the TST process and IGRA were calculated at around $2 and $20 respectively, cheaper than that reported elsewhere. The cost of individuals having to come back for TST reading can easily be offset by the comparatively high cost of the IGRA tests. TST and IGRA were shown to have moderate agreement in the study population, presenting it as a cheaper alternative when cost constitutes a limitation for TB control programs in developing countries [29,30].

This study showed that IGRA positivity and TST reactivity were associated with close exposure to index cases (IC) as evidenced by the significant differences in reactivity of IFN-γ release tests/TST among HHCs and CCs. These results are concordant with previous reports. The low prevalence of LTBI among CCs probably indicates lower than national average prevalence of TB in the study area. Sudan is a vast country and prevalence rates vary considerably between different counties [1].

In the absence of a gold standard test for the diagnosis of LTBI, developing countries with high burden of TB have been relying on TST to diagnose LTBI especially in children, where treatment of LTBI diagnosed by TST has been shown to reduce the risk of progression to active TB by 60-90% [31,32].

In this study IFN-γ release test results gave a high LTBI point prevalence that is comparable to that reported from India and Ethiopia [33,34].

Purified Protein Derivative (PPD) gave similar results to ESAT-6/CFP-10 ± TB7.7 in whole blood IFN-γ release assay, with an almost perfect degree of agreement, making it a cheap alternative to recombinant antigens. This is in concordance with previous reports by Brock and colleagues [32] where they reported comparable levels of sensitivities for the QuantiFERON-TB test based on *M. tuberculosis*-PPD and that based on ESAT-6 or CFP-10 antigens. These findings can markedly reduce the high cost of IFN-γ-release assay tests. However we didn’t find a significant difference in terms of PPD IFN-γ whole blood assays levels between HHCs and CCs, which may imply a significant overlap between the values in these BCG-vaccinated populations due to lower specificity of PPD.

## Conclusion

IFN-γ release assay (IGRA) using PPD or ESAT-6/CFP-10 give higher LTBI point prevalence compared to TST, which may indicate better sensitivity but the high cost of IGRA could be a concern for some developing countries. Although a less specific test, Purified Protein Derivative (PPD) gave comparable results to ESAT-6/CFP-10 ± TB7.7 in whole blood IFN-γ release assay, making it a cheap alternative to recombinant antigens.

## Abbreviations

LTBI: Latent TB Infection; TST: Tuberculin skin test; IFN-γ: Interferon-gamma; IGRA: Interferon gamma release assay.

## Competing interests

The authors declare that they have no competing interests.

## Authors’ contributions

AOS, EAG conceived and wrote the study proposal, prepared the necessary scientific and ethics approvals and contributed to volunteers’ recruitment, TST testing and wrote the manuscript. KAM, AOS carried the blood collections and the immunological tests. AMM, AAB, AHA, FMI & AME were involved in volunteers’ recruitment, care and follow-up, participated in manuscript writing and revision. All authors read and approved the final manuscript.

## Pre-publication history

The pre-publication history for this paper can be accessed here:

http://www.biomedcentral.com/1471-2458/13/1128/prepub

## References

[B1] WHO Report Global Tuberculosis Control 2010 WHO/HTM/TB/2010.7

[B2] Machado A Emodi K Takenami I Finkmoore BC Barbosa T Carvalho J Cavalcanti L Santos G Tavares M Mota M Barreto F Reis MG Arruda S Riley LW Analysis of discordance between the tuberculin skin test and the interferon-gamma release assay IJTLD 2009 13 446 453 19335949

[B3] Huebner RE Schein MF Bass JB Jr The tuberculin skin test Clin Infect Dis 1993 13 968 975 10.1093/clinids/17.6.968 8110954

[B4] Pai M Zwerling A Menzies D Systematic review: T-cell-based assays for the diagnosis of latent tuberculosis infection: an update Ann Intern Med 2008 13 177 184 10.7326/0003-4819-149-3-200808050-00241 18593687 PMC2951987

[B5] Dheda K Rook G Zumla A Peripheral T cell IFN-gamma responses and latent tuberculosis Am J Respir Crit Care Med 2004 13 97 98 15220126 10.1164/ajrccm.170.1.958

[B6] Barth RE Mudrikova T Hoepelman AIM Interferon-gamma release assay (IGRAs) in high-endemic settings: could they play a role in optimizing global TB diagnostics. Evaluating the possibilities of using IGRAs to diagnose active TB in a rural African setting Int J Infect Dis 2008 13 1 6 10.1016/j.ijid.2008.03.026 18565778

[B7] Shakak AO Khalil EAG Musa AM Salih KAM Bashir AA Ahmed AH Idris FM Elhassan AM Possible risk factors of progression to overt disease among individuals with latent tuberculosis infection in the Sudan IJCR 2013 13 1107 1110

[B8] Aggarwal A Dutta AK Timing dose of BCG vaccination in infants as assessed by post vaccination tuberculin sensitivity Indian J pediatr 1995 13 35 39 8613331

[B9] Cohen J A coefficient of agreement for nominal scales Educ Psychol Meas 1960 13 37 46 10.1177/001316446002000104

[B10] Ferebee SH Controlled chemoprophylaxis trials in tuberculosis. A general review Bibl Tuberc 1970 13 28 106 4903501

[B11] Sharma SK Mohanan S Sharma A Relevance of latent TB infection in areas of high TB prevalence Chest 2012 13 761 773 10.1378/chest.1377476 22948580

[B12] Lewinsohn DA Zalwango S Stein CM Mayanja-Kizza H Okwera A Boom WH Mugerwa RD Christopher C Whole blood interferon-gamma responses to mycobacterium tuberculosis antigens in young household contacts of persons with tuberculosis in Uganda PLoS ONE 2008 13 e3407 10.1371/journal.pone.0003407 18923705 PMC2560997

[B13] Diel R Loddenkemper R Neinhaus A Predictive value of interferon-γ release assays and tuberculin skin testing for progression from latent TB infection to disease state: a meta-analysis Chest 2012 13 63 75 Doi: 10.1378/chest.11-3157 10.1378/chest.1380697 22490872

[B14] Franken WPJ Timmermans JF Prins C Slootman EJ Dreverman J Bruins H Van Dissel JT Arend SM Comparison of Mantoux and QuantiFERON TB Gold Tests for Diagnosis of Latent Tuberculosis Infection in Army Personnel J Clin Vac Immunol 2007 13 477 480 10.1128/CVI.00463-06 PMC1865601 17301213

[B15] Diel R Goletti D Ferrara G Bothamley G Cirillo G Kampmann B Lange C Losi M Markova R Migliori GB Nienhaus A Ruhwald M Wagner D Zellweger JP Huitric E Sandgren A Manissero D Interferon-γ release assays for the diagnosis of latent Mycobacterium tuberculosis infection: a systematic review and meta-analysis Eur Respir J 2011 13 88 99 10.1183/09031936.00115110 21030451

[B16] Herrera V Perry S Parsonnet J Banaei N Clinical application and limitations of interferon-γ release assays for the diagnosis of latent tuberculosis infection Clin Infect Dis 2011 13 1031 1037 10.1093/cid/cir068 21460320

[B17] Sester M Sotgiu G Lange C Giehl C Girardi E Migliori GB Bossink A Dheda K Diel R Dominguez J Lipman M Nemeth J Ravn P Winkler S Huitric E Sandgren A Manissero D Interferon-γ release assays for the diagnosis of active tuberculosis: a systematic review and meta-analysis Eur Respir J 2011 13 100 111 10.1183/09031936.00114810 20847080

[B18] Brock I Weldingh K Lillebaek T Follmann F Andersen P Comparison of a new specific blood test and the skin test in tuberculosis contacts Am J Respir Crit Care Med 2004 13 65 69 10.1164/rccm.200402-232OC 15087297

[B19] Carvalho AC Pezzoli MC El-Hamad I Arce P Bigoni S Scarcella C Indelicato AM Scolari C Carosi G Matteelli A QuantiFERON-TB Gold test in the identification of latent tuberculosis infection in immigrants J Infect 2007 13 164 168 10.1016/j.jinf.2007.02.004 17428542

[B20] Diel R Nienhaus A Lange C Meywald-Walter K Forssbohm M Schaberg T Tuberculosis contact investigation with a new, specific blood test in a low-incidence population containing a high proportion of BCG-vaccinated persons Respir Res 2006 13 77 10.1186/1465-9921-7-77 16707012 PMC1481586

[B21] Poorhasan A Haghdoost M Mashrabi O Comparison of tuberculin skin test and interferon gamma assay for the diagnosis latent tuberculosis Am J Infect Dis 2010 13 6 50 53

[B22] Bellete B Coberly J Barnes GL Ko C Chaisson RE Comstock GW Bishai WR Evaluation of a whole-blood interferon-gamma release assay for the detection of Mycobacterium tuberculosis infection in study populations Clin Infect Dis 2002 13 1449 1456 10.1086/340397 12015690

[B23] Lighter J Rigaud M Eduardo R Peng C Pollack H Latent tuberculosis diagnosis in children by using the quantiFERON-TB Gold In-Tube test Pediatrics 2009 13 30 37 10.1542/peds.2007-3618 19117857

[B24] Black GF Weir RE Chaguluka SD Warndorff D Crampin AC Mwaungulu L Sichali L Floyd S Bliss L Jarman E Donovan L Andersen P Britton W Hewinson G Huygen K Paulsen J Singh M Prestidge R Fine PE Dockrell HM Gamma interferon responses induced by a panel of recombinant and purified mycobacterial antigens in healthy, non-Mycobacterium bovis BCG-vaccinated Malawian young adults Clin Diagn Lab Immunol 2003 13 602 611 12853392 10.1128/CDLI.10.4.602-611.2003 PMC164276

[B25] Fietta A Meloni F Cascina A Morosini M Marena C Troupioti P Mangiarotti P Casali L Comparison of a whole-blood interferon-gamma assay and tuberculin skin testing in patients with active tuberculosis and individuals at high or low risk of Mycobacteriumtuberculosis infection Am J Infect Control 2003 13 347 353 10.1016/S0196-6553(02)48240-5 14608301

[B26] Wrighton-Smith P Zellweger JP Direct costs of three models for the screening of latent tuberculosis infection Eur Respir J 2006 13 45 50 10.1183/09031936.06.00005906 16611659

[B27] Mahomed H Hughes EG Hawkridge T Minnies D Simon E Little F Hanekom WA Geiter L Hussey GD Comparison of Mantoux skin test with three generations of a whole blood IFN-γ assay for tuberculosis infection IJTLD 2006 13 310 316 16562712

[B28] Park YK Lee SH Kim SY Ryoo SW Kim CK Kim HJ Cho EH Yoo BH Lee J Ko W Interferon-γ release assay among tuberculin skin test positive students in Korean High Schools Tuber Respir Dis 2010 13 328 333 10.4046/trd.2010.68.6.328

[B29] Davies PDO Drobniewski F The use of interferon- γ based blood tests for the detection of latent tuberculosis infection Eur Respir J 2006 13 1 3 10.1183/09031936.06.00047606 16816343

[B30] Diel R Wrighton-Smith P Zellweger JP Cost-effectiveness of interferon-γ release assay testing for the treatment of latent tuberculosis Eur Respir J 2007 13 321 332 10.1183/09031936.00145906 17504793

[B31] Chadha VK Tuberculin Skin Test Indian J Pediatr 2001 13 53 58 10.1007/BF02728860 11237237

[B32] Brock I Munk ME Kik-Jensen A Andersen P Performance of whole blood IFN-gamma test for tuberculosis diagnosis based on PPD or the specific antigens ESAT-6 and CFP-10 Int J Tuberc Lung Dis 2001 13 462 467 11336278

[B33] Kabeer BSA Perumalb V Paramasivamc P Rajaa A Yield of QuantiFERON-TB gold in tube assay and tuberculin skin test in healthy persons from a tuberculosis endemic population J Pediatr Infect Dis 2011 13 125 129

[B34] Legesse M Ameni G Mamo G Medhin G Bjune G Abebe F Community-based cross-sectional survey of latent tuberculosis infection in Afar pastoralists, Ethiopia, using QuantiFERON-TB Gold In-Tube and tuberculin skin test BMC Infect Dis 2011 13 89 10.1186/1471-2334-11-89 21477326 PMC3080306

